# Combination of levofloxacin and cisplatin enhances anticancer efficacy via co-regulation of eight cancer-associated genes

**DOI:** 10.1007/s12672-022-00541-x

**Published:** 2022-08-19

**Authors:** Xiaoqiong He, ·Qian Yao, Dan Fan, Yutong You, Wenjing Lian, Zhangping Zhou, Ling Duan

**Affiliations:** 1 School of Public Health, Kunming Medical University, Kunming, 650500 Yunnan Province People’s Republic of China; 2 Institute of Yunnan Tumor, The Third Affiliated Hospital of Kunming Medical University, Kunming, 650118 Yunnan Province People’s Republic of China

**Keywords:** Levofloxacin, Cisplatin, Chemosensitizer, Combined chemotherapy, Drug repositioning

## Abstract

Chemosensitizer or combined chemotherapy can sensitize cancer cells to therapy and minimize drug resistance. We reveal that levofloxacin has broad-spectrum anticancer activity. Here we report that combination of levofloxacin and cisplatin further enhanced cytotoxicity in cancer cells by further promotion of apoptosis. Levofloxacin concentration-dependently promoted the inhibition of clone formation in cancer cells treated by cisplatin, and their combination further suppressed the tumor growth in mice. Levofloxacin and cisplatin co-regulated genes in directions supporting the enhancement of anticancer efficacy, of which, THBS1, TNFAIP3, LAPTM5, PI3 and IL24 were further upregulated, NCOA5, SRSF6 and SFPQ were further downregulated. Out of the 24 apoptotic pathways significantly enriched in the combination group, TNFAIP3, THBS1, SRSF6 and SFPQ overlapped in 14, 13, 3 and 1 pathway respectively. Jak-STAT/Cytokine-cytokine receptor interaction pathway network and extrinsic apoptotic signaling pathway were significantly enriched in levofloxacin group, cisplatin group and combination group. Jak-STAT/Cytokine-cytokine receptor interaction/Focal adhesion/EMC-receptor interaction pathway network was significantly enriched in the combination group, and IL24 and THBS1 were the overlapped genes. In conclusion, enhancement of anticancer efficacy in combination group was associated with the further regulation of THBS1, TNFAIP3, LAPTM5, PI3, IL24 and NCOA5, SFPQ, SRSF6. Targeting of Jak-STAT/Cytokine-cytokine receptor interaction/Focal adhesion/EMC-receptor interaction pathway network was correlated to the enhancement. With additional benefit to cancer patients for treatment or prophylaxis of an infectious syndrome, levofloxacin can benefit cancer chemotherapy no matter it is used independently or used with other chemotherapeutic drugs.

**Supplementary Information:**

The online version contains supplementary material available at 10.1007/s12672-022-00541-x.

## Introduction

Cancer is a leading death cause in the world and an enormous burden on society [1–3]. Chemotherapy is a mainstay treatment for cancers, but its use was limited because of toxic side effects and multidrug resistance that may necessitate the termination of treatment in some patients [4, 5]. Combined chemotherapy that potentiates cellular response to chemotherapeutic drug and reduce drug resistance can improve patient tolerance and response to therapy [6]. Chemosensitizer can not only augment the anticancer efficacy of the chemotherapeutic drugs by lowering the dose of killing cancer cells and reducing the incidence and severity of toxic side effects, but also delay/prevent the development of chemoresistance in cancers in adjuvant therapy. Sensitization of chemotherapeutic drugs and combined chemotherapy are regular practices in cancer therapy [7, 8].

Levofloxacin is an antibacterial agent by inhibiting DNA gyrase and topoisomerase IV in bacteria, which are essential for DNA transcription, duplication, repairing and recombination, and then consequently halting DNA replication [9]. Zusso et al. find that the anti-inflammatory activity of levofloxacin involves the activation of TLR4/NF-κB signaling pathway [10]. Levofloxacin increases the ratio of Bax/Bcl-2 and active caspase-3 in a dose-dependent manner [11]. Mondal et al. reported that some fluoroquinolones have antiproliferative activity and induced apoptosis in lung cancer cell line NCI-H460 in culture [12]. We reveal that levofloxacin has broad-spectrum anticancer activity in vitro and in vivo [13], in which, levofloxacin arrests cell cycle at G2/M phase, promotes apoptosis, and concentration-dependently regulates genes and signaling pathways favoring anticancer activity. Cisplatin (DDP) is a platinum-based DNA damaging agent, which is widely used in cancer chemotherapy. DDP prevents DNA duplication and DNA synthesis of cancer cells by inducing DNA-DNA cross-linkage and arresting cell cycle at G2/M phase [14]. Both levofloxacin and DDP prevent DNA duplication and DNA synthesis by preventing DNA from unwinding and arrest cell cycle at G2/M phase, but they act by different mechanisms. Infection is the most frequent and life-threatening complication in cancer patients [15, 16], and levofloxacin is often used for the treatment of bacterial infectious diseases. We question, with additional antibacterial benefit and anticancer activity, whether levofloxacin can potentiate the anticancer efficacy of chemotherapeutic drugs or levofloxacin can be used as a chemosensitizer in cancer chemotherapy.

## Materials and methods

### Samples

Levofloxacin hydrochloride (LH) injection was purchased from Community Hospital of Jinchen Street. DDP and 5-Fu (5-fluorouracil) were acquired from the Third Affiliated Hospital of Kunming Medical University. They were dissolved or diluted by PBS before use (stored in shadow at 4℃ fridge). In our study [13], LH show strong anticancer activity at the concentrations of 25–400 µg/ml in vitro. If LH is used for treatment of malignant ascites or pleural fluid by intraperitoneal perfusion or by thoracic perfusion, LH concentration in ascites or pleural fluid will be much higher than 200 µg/ml when it is used at the standard recommended dose (500 mg/day). Therefore, the final maximal concentration of LH in this study was 200 µg/ml in the in vitro assays. DDP concentration was 0.5, 1.0, 2.0, 3.0 and 4.0 µg/ml; and 5-Fu was 1.0, 2.0, 4.0, 8.0 and 16.0 µg/ml in the cell viability assay. LH and DDP were given to mice by intraperitoneal injection in tumor growth assay, and the dose of DDP was 2.0 mg/kg bw and LH was 50.0 mg/kg bw. For scientific comparison, study of this paper was carried out with the studies of other papers for the same assay at the same time to save the overlapped groups [13, 17].

### Cell lines and cell culture

HCT116 (human colorectal cancer cell line), CNE2 (human nasopharyngeal cancer cell line), HepG2 (human hepatocellular cancer cell line), A549 (human non-small cell lung cancer cell line), U251 (human neuroglioma cell line), XWLC05 (human Xuanwei lung cancer cell line), SGC-7901 (human stomach gastric cancer cell line), MCF7 (human breast cancer cell line) and K562 (human leukemia cell line) were used in the present study. These cell lines were provided by the Institute of Yunnan Tumor stocks, which were acquired from the Cell Bank of Kunming Animal Institute, Chinese Academy of Sciences. All cell lines for academic study are free of mycoplasmas and their STR profiling is validated by the cell bank. Cells were cultured in DMEM/F12 medium (Sigma) adding 10% fetal bovine serum (HyClone) at 37 °C in a 5% CO_2_, humidified incubator.

### Mice

6–8 week-age balb/c nude male mice were provided by Beijing Vital River Laboratory Animal Technology Co., Ltd. Mice were fed autoclaved water and rodent chow in the Specific Pathogen Free Animal Facility of Kunming Medical University, with a 12-h dark–light cycle. All animal procedures in this manuscript were approved by the Ethical Committee of Kunming Medical University, China. Animal study was in accordance with institutional and ARRIVE guidelines [18].

### Cell viability assay

MTT method was used to determine the cellular viability. Briefly, cells were collected in the logarithmic growth stage and were seeded into 96-well plates at a density of 6000 cells/well in 200 µl complete DMEM/F12 medium (MCF7 cells were 10,000 cells/well). After cells were cultured in the incubator overnight, the original medium in each well was replaced with 200 µl new medium containing samples. Cells were administrated with different concentrations of DDP (0, 0.5, 1.0, 2.0, 3.0 and 4.0 μg/ml) in LH-free medium (LH: 0 μg/ml, DDP group) or in LH medium (LH: 200 μg/ml, LH + DDP group) for 72 h respectively. 8 replicate wells were done in each group at the same time. 72 h later, the medium in each well was replaced with 200 µl new complete medium containing 10% MTT (5 mg/ml). Cells were cultured for 4 h in the incubator. After removing the MTT medium, adding 150 µl DMSO into each well, plates were shaken in shadow for 10 min. A microplate spectrophotometer reader at 490 nm detected the OD (optical density). Deleting the highest and the lowest OD values in each group, 6 OD values were retained for statistical analysis. For HepG2 and CNE2 cell lines, three independent experiments were carried out. Inhibition rates (IR) of cell viability were calculated.

$${\text{Inhibition}}\,{\text{rate}}\,\left( {{\text{IR}}} \right) = \left( {1 - {\text{Ad}}/{\text{Ac}}} \right) \times \,100\%$$

Ac: the corrected OD value of the negative control group; Ad: the corrected OD value of the drug administration group; Corrected OD value = OD value of sample group – OD value of blank group.

Evaluation of drug combination was determined by the following formula [19]:

$${\text{Combination}}\,{\text{index}}\,\left( {\text{Q}} \right) = {\text{E}}_{{\left( {{\text{A}} + {\text{B}}} \right)}} /\left( {{\text{E}}_{{\text{A}}} + {\text{E}}_{{\text{B}}} - {\text{E}}_{{\text{A}}} \times {\text{E}}_{{\text{B}}} } \right)$$

E_(A+B)_: IR of the combination of drug A and drug B; E_A_: IR of drug A; E_B_: IR of drug B; Antagonistic effect: Q ≤ 0.85. Additive effect: Q = 0.85–1.15. Synergistic effect: Q ≥ 1.15.

### Plate clone formation assay

1000 cells were added into each well of 6-well culture plates and cultured overnight in a 37℃ incubator. On the next day, medium in each well was replaced with 2 ml of complete medium containing different drugs. Cells were treated with DDP (0, 0.1, 0.2, 0.4, 0.8 and 1.6 μg/ml) in LH-free medium (LH: 0 μg/ml, DDP group) or in LH medium (LH: 50 μg/ml or LH: 100 μg/ml, LH + DDP group) for 14 days respectively. 3 replicate wells were done in each group. During the period, medium was renewed on the 5th, 9th and 12th day while checking colony and cell morphology. After removing the medium in each well on the 14th day, each well was washed with 2 ml PBS for one time and added 1 ml 5% paraformaldehyde to fix for 15 min. Wells were then washed with PBS once, stained with 0.5% crystal violet for 10 min, and washed again with PBS before taking pictures with a camera. The colony number was counted. The inhibition rate (IR) of colony number was calculated by following formula:

${\text{Inhibition}}\,{\text{rate}}\,\left( {{\text{IR}}} \right) = \left( {1 - {\text{Nd}}/{\text{Nc}}} \right) \times \,100\%$Nc: colony number of the negative control group; Nd: colony number of the drug administration group.

### Cell cycle and apoptosis detection by flow cytometry

CNE2 and HepG2 cells were treated with PBS (NC group), LH (100 µg/ml, LH group), DDP (2 µg/ml, DDP group) or LH + DDP (DDP: 2 µg/ml and LH: 100 µg/ml, LH + DDP group) for 48 h. Apoptosis was examined by Annexin-V FITC/PI kit according to the protocol. Floating and adherent cells were completely collected. Cells of each sample were resuspended with 0.4 ml binding buffer. Adding 5 μl Annexin-V FITC into each sample. Samples were incubated on ice for 15 min. Each sample was added 10 μl PI and incubated for 30 min at room temperature in the shadow before flow cytometry detection (> 10,000 cells/sample). Viable cells were Annexin-V FITC negative and PI negative, early apoptotic (EA) cells were PI negative but Annexin-V FITC positive, late apoptotic (LA) cells were Annexin-V FITC positive and PI positive, and mechanically damaged and necrotic cells were PI positive but Annexin-V FITC negative.

In the detection of cell cycle position, CNE2 cells were collected after being administrated with PBS (NC group), DDP (2 µg/ml, DDP group), LH (200 µg/ml, LH group) or LH + DDP (LH: 200 µg/ml and DDP: 2 µg/ml, LH + DDP group) for 48 h. The collected cells were carefully resuspended into 5 ml of 70% ethanol on a mixer and fixed overnight in a 4 ℃ fridge. On the next day, cells were centrifuged for 5 min at 1000 rpm. Discarding the fixative, cells were washed with 2 ml PBS for two times, then resuspended with 500 μL of PI/RNase solution and stained in the dark at room temperature for 15 min. More than 10,000 cells were analyzed for each sample.

### Tumor growth assay

CNE2 cells were collected and rinsed with 0.01 M PBS twice and then resuspended with cooled DMEM/F12 medium (free of antibiotics and FBS) to 1 × 10^7^ cells /ml. 0.1 ml cell suspension was injected subcutaneously on the right-side flank of mouse. 8 days after inoculation, the qualified tumor-bearing mice (tumor volume was 100 ~ 300 mm^3^) were randomly allocated into 4 groups (6 tumor-bearing mice in each group) according to tumor volume (TV). Mice in negative control group were injected neutral saline (NS), in LH group were injected LH of 50 mg/kg bw, in DDP group were injected DDP of 2 mg/kg bw and mice in LH + DDP group were injected both LH (50 mg/kg bw) and DDP (2 mg/kg bw) at half hour interval. LH or NS were injected intraperitoneally twice a day at 6 h interval (9:00 Am and 15:00 Pm), 0.10 ml /10 g bw per injection. DDP was given in the morning, once a day at one day interval. TV and tumor-bearing body weight (BW) of each mouse were recorded every 4 days. Tumor size was measured by the same operator using an auto-reading caliper throughout the study.

The maximum diameter of tumor in adult mouse suggested in “guidelines for endpoints in animal study proposals” by NIH (National Institute of Health, USA) in 2019 is 20 mm. The Ethical Committee of Kunming Medical University refers to this guideline. CNE2 xenografts grew rapidly in mice in the neutral saline control group. At the second examination point (8 days of drug administration), the maximum diameter of a few tumors was close to 20 mm. Because shorter time of drug administration could not show the efficacy of drugs, we got permission from the Ethical Committee of Kunming Medical University to end our animal study at the third examination point (12 days of drug administration). On the 12^th^ day of drug treatment, tumors were isolated and weighed after mice were euthanized.

TV (tumor volume) = a × b^2^/2. “a” indicates the longitude span of tumor; “b” indicates the short diameter of tumor.

RTV (relative tumor volume) = V_T_/V_0_. V_0_ is the TV measured before drug administration, V_T_ is the TV measured during drug administration.

RPR (relative proliferation rate) = T_RTV_/C_RTV_ × 100%. T_RTV_ is the RTV of the drug group, C_RTV_ is the RTV of the NS group.

### Microarray gene expression profiling

CNE2 cells were intervened with PBS (NC group), DDP (2 µg/ml, DDP group), LH (100 µg/ml, LH group) or LH + DDP (LH: 100 µg/ml and DDP: 2 µg/ml, LH + DDP group) for 48 h. Total RNA was extracted and used for transcriptome profiling (Affymetrix GeneChip^®^ Human Transcriptome Array 2.0). Shanghai Qi Ming Biological Information LTD was responsible for microarray gene expression profiling analysis. Gene expression data was analyzed by experts using Gene Cloud Biotechnology Information (GCBI) software. Robust Multiarray Average method was used for the pre-analysis of Gene chip data. Gene Ontology (GO) and Kyoto Encyclopedia of Genes and Genomes (KEGG) were used in gene expression enrichment analyses. The genes were filtered using microarray fold change (FC) value of logFC ≥  ± 1.00.

### Quantitative reverse transcription PCR (RT–qPCR)

Seven further regulated genes in microarray data were validated by profiling the expression level through RT–qPCR. Each data point offered for the RT–qPCR assay was resulted from three biological replicates (BR). CNE2 cells were treated with DDP (2 µg/ml, DDP group), LH (100 µg/ml, LH group) or LH + DDP (LH: 100 µg/ml and DDP: 2 µg/ml, LH + DDP group) for 48 h. Total RNA was reversed transcribed and the cDNA from each BR was used as a template for the qPCR. Primers of the genes were synthesized by Shanghai Qi Ming Biological Information LTD (Supplementary Table S5). GAPDH was used as an internal reference to normalize the gene expression data. The cycling conditions were 95 °C for 30 s, followed by 40 cycles of 95 °C for 10 s and 60 °C for 30 s. Relative quantification of the selected genes was calculated using the 2^−△△CT^ method.

### Statistical analysis

Data was expressed as mean ± SD in duplicate assays. All data were analyzed using SPSS statistical software version 21.0. The data from three independent groups were analyzed by one-way ANOVA. Statistical comparison was carried out by independent *t*-test. ^*/#^*p* < 0.05, ^**/##^*p* < 0.01, and ^***/###^*p* < 0.001. The threshold for statistical significance was defined as *p* < 0.05.

## Results

### Combination of levofloxacin and cisplatin enhances the cytotoxicity in cancer cells

**Fig.1:**
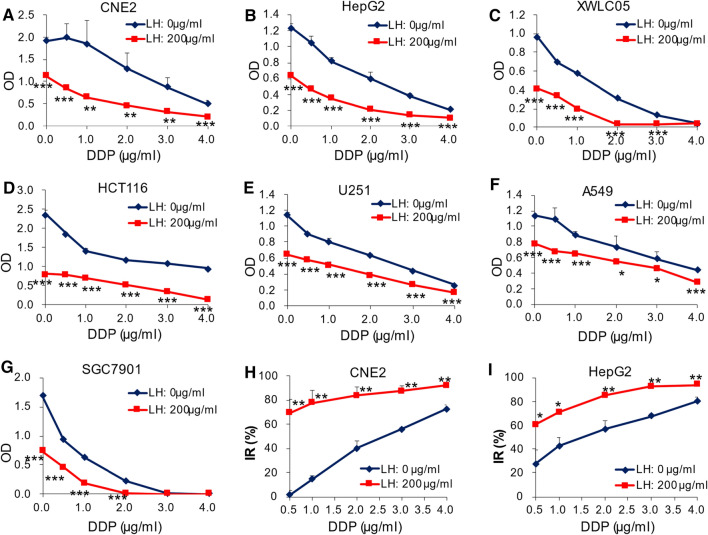
Combination of levofloxacin and cisplatin enhances cytotoxicity in cancer cells. **A–G** Comparation of OD between LH + DDP group and DDP group at each DDP concentration. CNE2 cells (**A**), HepG2 cells (**B**), XWLC05 cells (**C**), HCT116 cells (**D**), U251 cells (**E**), A549 cells (**F**), SGC7901 cells (**G**). **H** IR comparison between LH + DDP group and DDP group at each DDP concentration in CNE2 cells. **I** IR comparison between LH + DDP group and DDP group at each DDP concentration in HepG2 cells. Data is expressed as mean ± standard deviation (SD), A-G is from 6 replicates in one experiment, H and I is from 3 independent experiments. *p < 0.05, **p < 0.01 and ***p < 0.001

Levofloxacin hydrochloride (LH) shows universal antiproliferation activity in all cancer cell lines in our previous study (Fig. S1A) [13]. The combination cytotoxicity of LH + DDP was evaluated in eight human cancer cell lines (HepG2, A549, XWLC05, SGC7901, HCT116, CNE2, U251 and MCF7). Cell viability was measured after cancer cells were intervened with series concentrations of DDP (0, 0.5, 1.0, 2.0, 3.0 and 4.0 µg/ml) in LH-free medium (LH: 0 µg/ml, DDP group) or LH medium (LH: 200 µg/ml, LH + DDP group) for 72 h. As a cytotoxic anticancer agent, DDP concentration-dependently reduced the cell viability in all tested cancer cell lines in LH-free medium (Fig. 1A–G, LH: 0 µg/ml). While the medium containing 200 µg/ml of LH, compared with LH-free medium (DDP group), OD (optical density) in LH + DDP group in all cancer cell lines at each DDP concentration was significantly reduced (Fig. 1A–G, LH: 200 µg/ml) (*p* < 0.01 or 0.001). IR (inhibitory rate) of cell viability in CNE2 cells and HepG2 cells treated by LH + DDP were significantly promoted (data from 3 independent experiments, *p* < 0.05 or 0.01), compared with those in DDP group at each DDP concentration (Fig. 1H, I). LH + DDP significantly enhanced the cytotoxicity, but the enhancements varied in different cancer cell lines. Q values assessed by the evaluation of drug combination index indicated that combination of LH and DDP showed additive or synergistic effects in all cancer cell lines (Table 1).

**Table 1:** Q values in different cancer cell lines by the evaluation of drug combination

Cell line	DDP (µg/ml)				
	0.5	1.0	2.0	3.0	4.0
CNE2	1.42	1.52	1.26	1.13	1.05
XWLC05	0.94	1.07	1.11	1.02	0.98
A549	1.17	0.93	0.93	0.92	1.02
HepG2	1.11	1.09	1.11	1.06	1.01
HCT116	0.91	0.88	0.93	1.01	1.08
SGC7901	0.96	1.06	1.05	1.00	1.00
U251	0.90	0.92	0.97	0.99	0.98
MCF7	1.26	0.92	0.86	0.83	0.82

In addition to DDP, combination of LH and 5-Fu also significantly enhanced cytotoxicity in cancer cells (Fig. S1B).

Antagonistic effect: Q ≤ 0.85

Additive effect: 0.85 < Q < 1.15

Synergistic effect: Q ≥ 1.15

Q values indicate the combination effects

### Combination of levofloxacin and cisplatin further inhibits clone formation of cancer cells

**Fig. 2:**
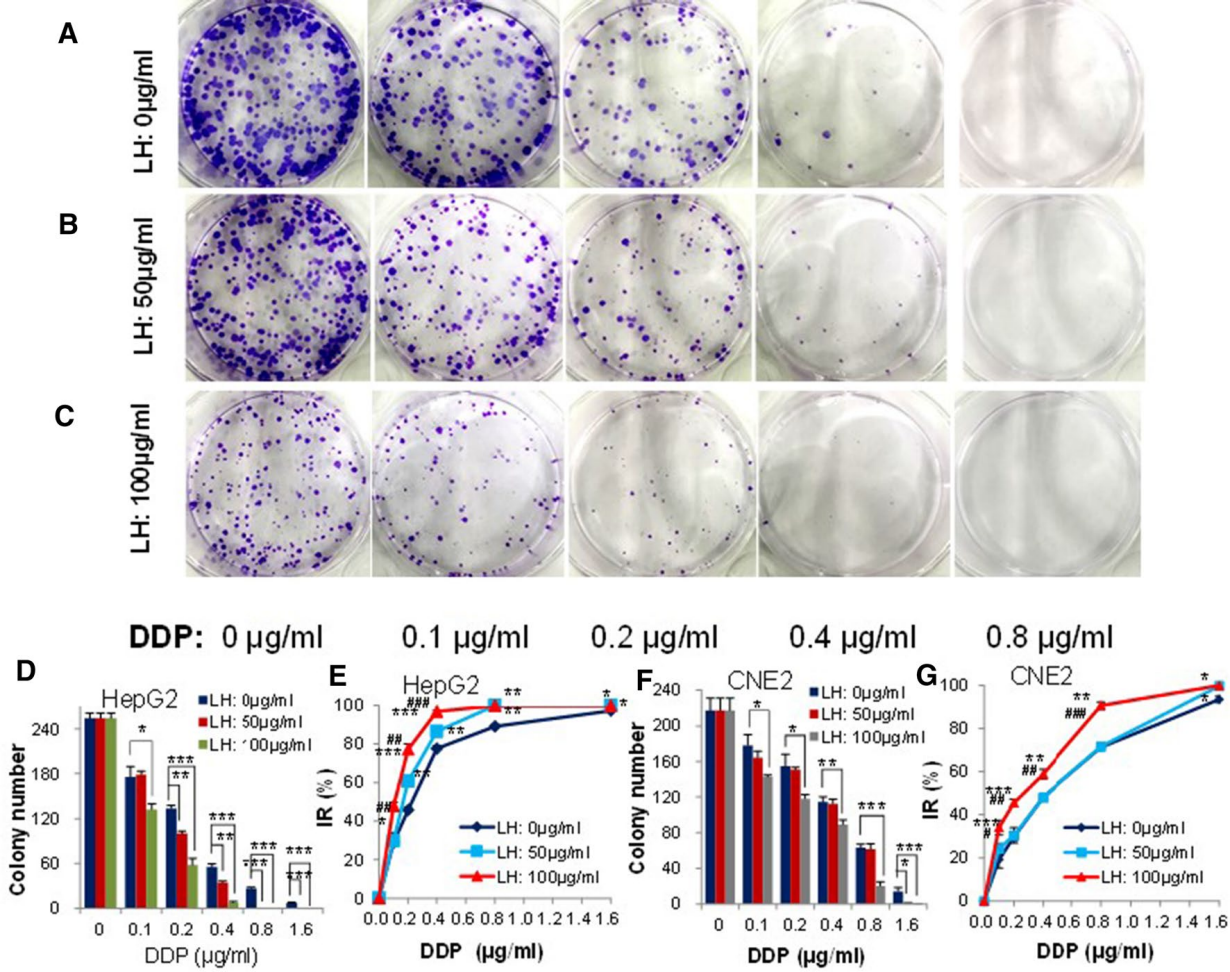
Levofloxacin concentration-dependently enhances the inhibition of clone formation of cancer cells treated by cisplatin. **A–C** Example of cloned colony in HepG2 cells treated by different concentrations of DDP in different concentrations of LH medium (0, 50 and 100 μg/ml). **D** Summary data of the colony number in HepG2 cells treated by different concentrations of DDP in different concentrations of LH medium (0, 50 and 100 μg/ml). **E** IR comparison among different concentrations of LH medium (0, 50 and 100 μg/ml) in HepG2 cells treated by different concentrations of DDP. **F** Summary data of the colony number in CNE2 cells treated by different concentrations of DDP in different concentrations of LH medium (0, 50 and 100 μg/ml). **G** IR comparison among different concentrations of LH medium (0, 50 and 100 μg/ml) in CNE2 cells treated by different concentrations of DDP. Error bars indicate SD from three independent experiments. *^/#^*p* < 0.05, **^/##^*p* < 0.01 and ***^/###^*p* < 0.001. *: comparison between LH-free medium (LH: 0 μg/ml) and LH medium (LH: 50 μg/ml or 100 μg/ml) at the same DDP concentration. ^#^: IR comparison between LH medium (LH: 50 μg/ml) and LH medium (100 μg/ml) at the same DDP concentration. IR: Percentage inhibition rate of colony numbers

The effects of different concentrations of LH on the clone formation of two cancer cell lines (HepG2 and CNE2) treated with series concentrations of DDP (0, 0.1, 0.2, 0.4, 0.8 and 1.6 μg/ml) were investigated. DDP alone obviously inhibited clone formation of two cancer cell lines in LH-free medium (LH: 0 µg/ml), and the colony number decreased with the increasing of DDP concentration (Fig. 2A). When medium contained 50 μg/ml (Fig. 2B) or 100 μg/ml (Fig. 2C) of LH, the clone formation of the cancer cells treated by DDP at each DDP concentration was further inhibited. Quantitatively, with the increasing of LH (0, 50, 100 µg/ml), the colony number that grew for HepG2 (Fig. 2D) and CNE2 (Fig. 2F) significantly decreased (*p* < 0.05, 0.01 or 0.001) and the IR of colony number in HepG2 (Fig. 2E) and CNE2 (Fi. 2G) significantly increased (*p* < 0.05, 0.01 or 0.001) at the same DDP concentration. The colony size in LH + DDP groups was obviously smaller than that in the DDP group and gradually decreased with the increasing of LH concentration, which meant that fewer cancer cells proliferated in the colonies in LH + DDP group. Compared with CNE2 cells, HepG2 cells were more sensitive to DDP no matter medium contained LH or not, which was consistent with the results of cell viability assay. When medium containing LH, no cancer cells survived and no colony was found in HepG2 when DDP concentration was more than 0.8 μg/ml, or in CNE2 when DDP concentration was 1.6 μg/ml. LH concentration-dependently enhanced the anticancer efficacy of DDP in both cancer cell lines.

### Levofloxacin and cisplatin arrest cell cycle at G2/M, and their combination enhances apoptosis of cancer cells

**Fig. 3:**
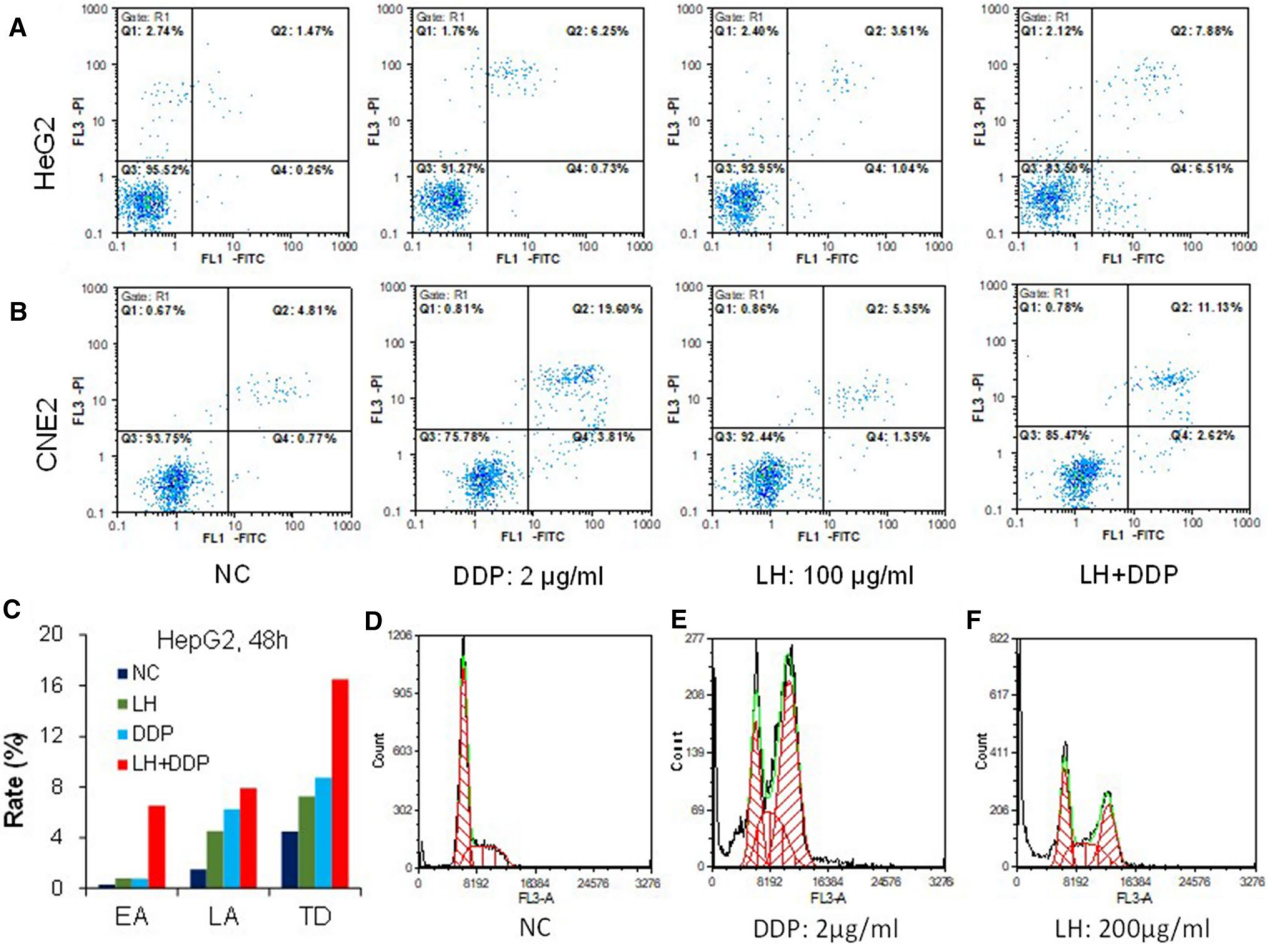
Effects of levofloxacin, cisplatin and their combination on the cell cycle and apoptosis of cancer cells. **A** FACS analysis of apoptosis in HepG2 cells. **B** FACS analysis of apoptosis in CNE2 cells. **C** Summary data of EA, LA and TD in different groups in HepG2 cells. **D** FACS analysis of cell cycle in negative control group. **E** FACS analysis of cell cycle in DDP group. **F** FACS analysis of cell cycle in LH group

Apoptosis was detected by flow cytometry after HepG2 cells and CNE2 cells were treated with DDP, LH, and LH + DDP for 48 h respectively (Fig. 3A, B). EA (early apoptosis, Q4), LA (later apoptosis, Q2) and TD (total death, Q1 + Q2 + Q4) in LH + DDP group in HepG2 cells were 6.51%, 7.88% and 16.51% respectively; which were much higher than those in LH group (0.77%, 4.52% and 7.26%) or in DDP group (0.73%, 6.25% and 8.74%) (Fig. 3C). Compared with LH group and DDP group, LH + DDP further enhanced apoptosis of cancer cells.

Cell cycle of CNE2 cells treated by DDP, LH, and LH + DDP for 48 h was also detected by flow cytometry. Compared with the control group (Fig. 3D), both DDP (Fig. 3E) and LH (Fig. 3F) arrested cell cycle at G2/M phase. The cell cycle in LH + DDP group could not be detected because little cells survived resulting from the severe cytotoxicity in this group. Evident cell fragment peak appeared in front of G0/G1 peak in LH group and DDP group, which meant that both LH and DDP induced cell damage and consequently cell fragments. Cell cycle arrest and apoptosis induction were the chemotherapeutic strategies of LH + DDP against cancers, which supports the enhancement of anticancer efficacy.

### Combination of levofloxacin and cisplatin further suppresses tumor growth in xenograft mouse model

**Fig. 4:**
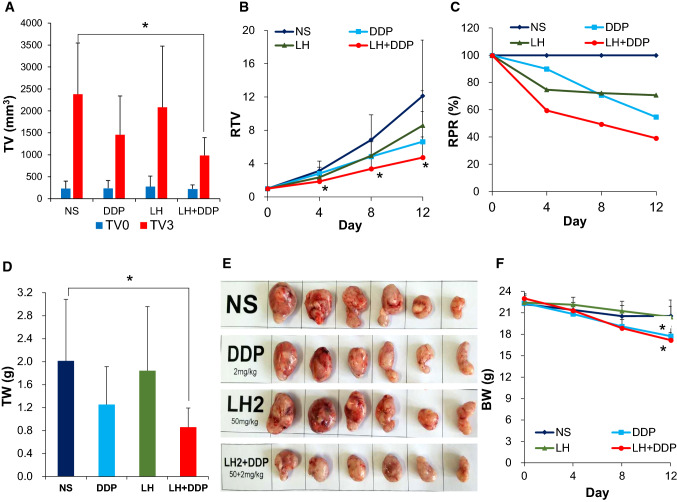
Effects of levofloxacin, cisplatin and their combination on the tumor growth in xenograft mouse model. **A** Tumor volume before drug administration (TV0) and at the end of the study (TV3). **B** RTV throughout the study. **C** RPR throughout the study. **D** TW at the end of the study. **E** The ex vivo tumor photos in different groups. **F** BW throughout the study. Data are mean ± SD from 6 mice [13]. Compared with NS group: *p < 0.05

The in vivo anticancer efficacy of LH, DDP and LH + DDP was compared in CNE2 xenograft mouse model. Mice were intraperitoneally injected with NS, DDP (2 mg/kg bw), LH (50 mg/kg bw) and LH + DDP respectively. TV0 (tumor volume before drug administration) in each group had no statistically significant difference (Fig. 4A) (*p* > 0.05). As a broad-spectrum anticancer drug, DDP alone obviously inhibited tumor growth although no significance was obtained because of short term of drug administration (6 times in 12 days) (Fig. 4B, C).

CNE2 xenografts grew rapidly in the neutral saline control group. At the third examination point (on the 12th day of drug administration), tumor volume in a few mice in NS group and in LH group exceeded 2000 mm^3^. We had to end animal study at this examination point. Ethical Committee of Kunming Medical University approved the procedure.

After 12 days of drug treatment, the final tumor volume (TV3) in LH + DDP group was 984.78 mm^3^, significantly less than 2380.64 mm^3^ in NS group (*p* < 0.05), and much less than 1457.36 mm^3^ in DDP group (*p* > 0.05) and 2082.96 mm^3^ in LH group (*p* > 0.05) (Fig. 4A). RTV (relative tumor volume) were 1.86-, 3.38- and 4.74-fold of TV0 at day 4, 8 and day 12 in LH + DDP group, which were significantly less than 3.12-, 6.83- and 12.11-fold in NS group (*p* < 0.05); and much less than 2.81-, 4.83- and 6.63-fold in DDP group and 2.34-, 4.94 and 8.57-fold in LH group respectively (Fig. 4B). RPR (relative proliferation rate of tumor) in LH + DDP group were 59.57%, 49.47% and 39.16% at day 4, 8 and 12, relative to the NS group. RPR in LH + DDP group sharply decreased with treating time and were much less than those in NS group, LH group, or DDP group throughout the study (Fig. 4C). Compared with NS group, TV (tumor volume) in LH + DDP group increased slowly since drug administration. Tumor growth inhibition of LH + DDP was better than that of LH or DDP alone (Fig. 4A, B). At the end of the experiment, TW (tumor weight) in LH + DDP group was 0.86 g, significantly less than 2.01 g in NS group (*p* < 0.05), and much less than1.25 g in DDP group and 1.84 g in LH group (Fig. 4D), which can also be judged by the ex vivo tumor size (Fig. 4E). Results confirmed that LH, DDP and LH + DDP could inhibit tumor growth, and the anticancer efficacy of LH + DDP was much better than LH or DDP alone. Compared with NS group, BW (body weight) of mice in DDP group and in LH + DDP group fell significantly after 12 days of drug administration (Fig. 4F) (*p* < 0.05), but LH did not show evident toxicity. Furthermore, BW in DDP group and in LH + DDP group were very close throughout the study (*p* > 0.05). The drop of BW in LH + DDP group was obviously resulted from the toxicity of DDP. LH + DDP promoted the anticancer efficacy in xenograft mice model without promoting the toxicity in mice.

### Combination of levofloxacin and cisplatin regulates genes supporting the enhancement of anticancer efficacy

To determine the mechanism by which combination of LH and DDP enhances anticancer efficacy, we investigated potential changes in gene expression by microarray assay. RNA was extracted from CNE2 cells treated with PBS, DDP, LH and LH + DDP for 48 h.

**Fig. 5:**
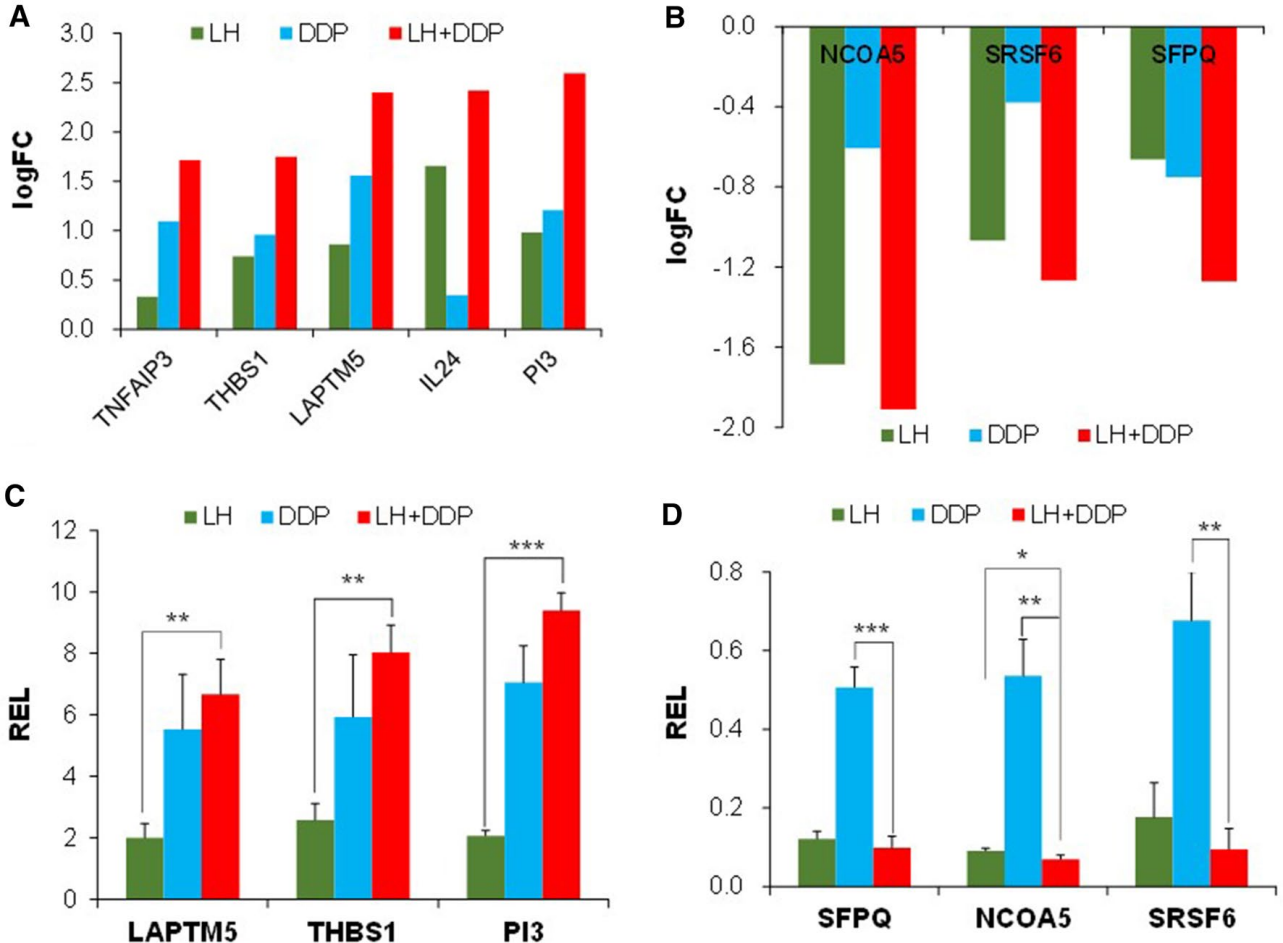
Combination of levofloxacin and cisplatin regulates genes in direction supporting the enhancement of anticancer efficacy. **A** LogFC of the upregulated differential genes. **B** LogFC of the downregulated differential genes. **C** REL of the upregulated differential genes. **D** REL of the downregulated differential genes. REL = 1 in PBS control group. Data are expressed as mean ± SD from 3 biological replicates in RT–qPCR. Compared with PBS control group: *p < 0.05, **p < 0.01, ***p < 0.001

168, 178, and 206 transcripts were noted to be differentially regulated in LH group, DDP group and LH + DDP group using a cut-off logFC (fold change) ≥  ± 1.00, with 18, 40 and 121 up-genes respectively. Compared with LH group and DDP group, the differential up-genes favoring anticancer activity and their logFC being further enhanced in LH + DDP group were PI3, THBS1, LAPTM5, TNFAIP3, and IL24 (Fig. 5A); the differential down-genes favoring anticancer activity and their logFC being further reduced in LH + DDP group were NCOA5, SFPQ and SRSF6 (Fig. 5B). Expressions of these genes in LH + DDP group were further upregulated or downregulated in direction supporting the promotion of anticancer efficacy. Co-regulation of the above 8 differential genes in LH + DDP group likely contributed to the enhancement of anticancer efficacy. These genes might be the combination chemotherapeutic targets of LH + DDP against cancers.

The validation was carried out by measuring the expression levels of 7 genes (TNFAIP3, THBS1, PI3, LAPTM5, SFPQ, SRSF6, NCOA5) through RT–qPCR, to assess if LH enhances the anticancer efficacy of DDP by regulating the differential genes in DDP group. IL24 was not validated because its logFC in LH group was much higher than that in DDP group and IL24 was not a differential gene in DDP group. Results showed that, out of the 7 validated genes, REL (relative expression level) of 6 genes (PI3, THBS1, LAPTM5 and NCOA5, SFPQ, SRSF6) agreed with the tendency of logFC, in which their REL were further upregulated or down-regulated in LH + DDP group in direction supporting the promotion of anticancer efficacy (Fig. 5C, D). REL of the further upregulated differential genes in LH + DDP group were higher than the REL in LH group (*p* < 0.01 or 0.001) and the REL in DDP group. REL of the further downregulated differential genes in LH + DDP group were less than the REL in DDP group (*p* < 0.01 or 0.001) and the REL in LH group. These 6 differential genes might be the combination chemotherapeutic targets for the enhancement of anticancer efficacy in LH + DDP group. REL of TNFAIP3 were 1.527, 5.967 and 3.459 in LH group, DDP group and LH + DDP group respectively. Although TNFAIP3 overexpressed in LH + DDP group, compared with DDP group, REL of TNFAIP3 was not further enhanced in LH + DDP group.

### Combination of levofloxacin and cisplatin regulates signaling pathways and pathway networks associating with anticancer activity

Microarray gene expression profiling revealed that LH + DDP significantly regulated canonical pathways that are associated with DNA replication, cell proliferation, cell cycle, and apoptosis. 8, 19 and 24 apoptotic pathways (Biological Pathway in GO-term) were significantly enriched in DDP group, LH group and LH + DDP group respectively (Table S1, S2). Out of the 24 apoptotic pathways in LH + DDP group, 15 pathways were also significantly enriched in LH group, and 2 pathways were enriched in DDP group. 9 new apoptotic pathways appeared in LH + DDP group, in which, 6 apoptotic pathways (GO: 2,001,237, 0,001,783, 0,008,625, 0,043,277, 0,003,278 and 0,043,652) were regulated by the differential up-genes and 3 apoptotic pathways (GO: 2,000,674, 1,902,177 and 0,097,050) were regulated by the differential down-genes. THBS1, TNFAIP3, SFPQ and SRSF6 were the overlapped genes in the 9 new apoptotic pathways. Extrinsic apoptotic signaling pathway (GO: 2,001,236) was the only apoptotic pathway enriched in LH group, DDP group and LH + DDP group at the same time, and THBS1 and TNFAIP3 were the overlapped gene of this pathway in LH + DDP group. In the 24 significantly enriched apoptotic pathways in LH + DDP group, TNFAIP3, THBS1, SFPQ and SRSF6 overlapped in 14, 13, 3 and 1 apoptotic pathways respectively. Further regulation of these genes likely contributed to the enhancement of apoptosis in combination group. 20 up-gene-goPathways (Table S3) and 5 down-gene-goPathways (Table S4) were significantly enriched in LH + DDP group by KEGG.

**Fig. 6:**
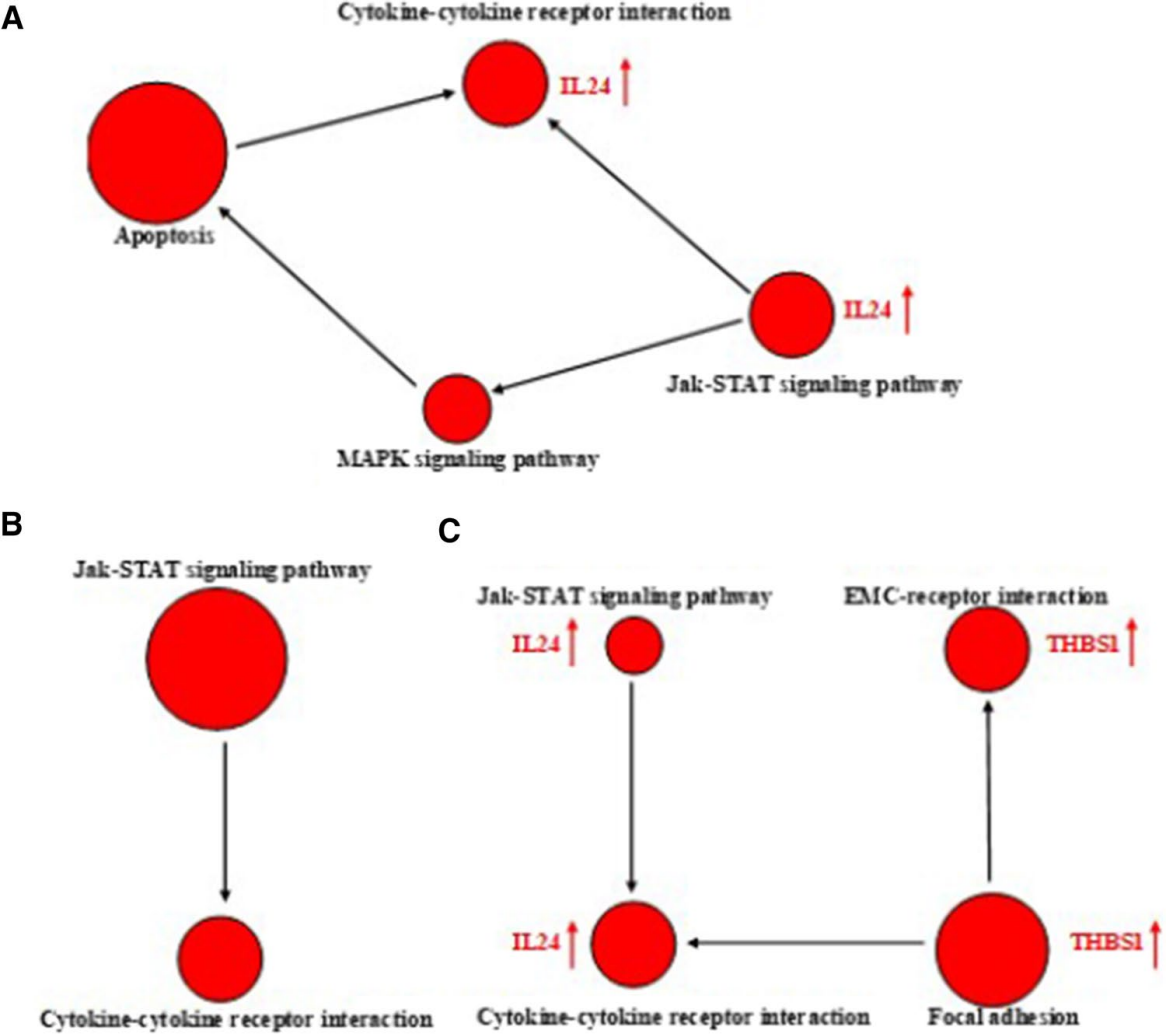
The signaling pathway networks significantly enriched by KEGG. **A** Signaling pathway network enriched in LH group. **B** Signaling pathway network enriched in DDP group. **C** Signaling pathway network enriched in LH + DDP group

LH, DDP and LH + DDP regulated signaling pathway networks associating with anticancer activity. Jak-STAT/Cytokine-cytokine receptor interaction pathway network was the commonly enriched pathway network in LH group, DDP group and LH + DDP group (Fig. 6). IL24 was the top one upregulated differential gene overlapping in the two signaling pathways in this network in LH group and LH + DDP group (Fig. 6A, C). Focal adhesion/ECM-receptor interaction pathway/Jak-STAT / Cytokine-cytokine receptor interaction pathway network was the significantly enriched pathway network in LH + DDP group, and THBS1 and IL 24 were the upregulated differential gene in the pathway network (Fig. 6C). As the expression levels of IL24 and THBS1 were further upregulated in LH + DDP group, IL24 and THBS1 may contribute to the enhancement of anticancer efficacy by the above pathway network.

GO analysis showed that 584 signaling pathways were significantly enriched in LH + DDP group, and THBS1, TNFAIP3 and PI3 overlapped in 150, 94 and 12 biological pathways (BP, Go-term) respectively. Out of the 70 significantly enriched molecular functions (MF, GO-term), 25 were associated with the regulation of molecule binding, and THBS1 and TNFAIP3 overlapped in 11 and 1 molecule binding functions. All the top ten cell components (CC, GO-term) and the top ten biological pathways enriched in the Downgene-Sig-Go were associated with the regulation of genetic substances. Top ten cell components, biological pathways and molecular functions (GO-term) significantly enriched in LH + DDP group are shown in Supplementary Figure S2.

## Discussion

Lots of approaches and strategies are employed to overcome the adverse effects in cancer chemotherapy, in which sensitization of anticancer drugs and combined chemotherapy are the regular practices. The relation between the treatment of antibiotics and the anticancer activity of anticancer drug is currently hot topic. Several previous studies revealed the improvement of treatment efficacies of chemotherapeutic drugs by the addition of antibiotics in the patients with advanced cancer [20–23]. In our previous study [13], we show levofloxacin exerts broad-spectrum anticancer activity via regulation of THBS1, LAPTM5, SRD5A3, MFAP5 and P4HA1. In the present study, we first found that levofloxacin could enhance the cytotoxicity of cisplatin in all cancer cell lines in a concentration-dependent manner. Both levofloxacin and cisplatin arrested cancer cells at G2/M phase. Combination of levofloxacin and cisplatin promoted apoptosis induction in cancer cells. Moreover, their combination significantly potentiated anticancer efficacy in vivo. Regulation of THBS1, TNFAIP3, LAPTM5, PI3, IL24, SFPQ, SRSF6 and NCOA5 might contribute to the enhancement of anticancer efficacy in combination group. Our study highlights that levofloxacin can potentiate the anticancer efficacy of chemotherapeutic drugs in the context of combined regimens. With additional benefit for treatment or prophylaxis of an infectious syndrome, levofloxacin can benefit cancer therapy no matter it is used independently or used with other chemotherapeutic drugs at the same time.

Elafin (PI3) is transcriptionally downregulated in most cancer cell lines [24]. PI3 suppresses tumor growth via the inhibition of elastase [25]. PI3 induces apoptosis in melanoma cells by intrinsic apoptotic pathway, and suppression of PI3 in melanoma leads to disease progression [26]. PI3 was the differential up-gene both in DDP group and LH + DDP group, and its expression level in LH + DDP group was further promoted. PI3 overlapped in 12 significantly regulated biological pathways in LH + DDP group. Most of the pathways are associated with the regulation of peptidase, endopeptidase activity and epidermal cell differentiation, which may contribute to anticancer activity.

Thrombospondin 1 (THBS1) is known to be antiangiogenic which could inhibit vascularization in tumors. THBS1 is an anti-oncogene which plays an essential role in the tumor microenvironment. THBS1 is involved in the modulation of many carcinogenesis-associated processes, such as angiogenesis, apoptosis, cell–cell adhesion, invasion, proliferation, chemoresistance, motility and migration [27]. THBS1 is a tumor suppressor in lung carcinoma [28]. Suppression of THBS1 enhances the progression, invasion, and migration of bladder cancer [29]. Chan et al. find that THBS1 abundance in the exosomes of nasopharyngeal carcinoma cells is reduced, and point out that the essential roles of THBS1 in the exosomes-induced angiogenesis might be the therapeutic targets in future [30]. THBS1 is the chemotherapeutic target of many conventional chemotherapeutic drugs. 5-Fu promoted the expression level of THBS1 in human breast cancer cells and colorectal carcinoma cells in a dose-dependent manner. 5-FU–based drugs exert antitumor function and antiangiogenic function through upregulation of THBS1 [31]. Bocci et al. reveal that THBS1 overexpression induced by cyclophosphamide is associated with the suppression of tumor growth [32]. In the present study, THBS1 overexpressed in LH group, DDP group and LH + DDP group which favored the anticancer activity. THBS1 expression level was further enhanced by LH + DDP which supported the enhancement of anticancer efficacy in this group. THBS1 was the most frequently overlapped gene in the 584 significantly regulated biological pathways (BP, GO-term) in LH + DDP group, in which THBS1 overlapped in 150 biological pathways, especially in 13 of the 20 significantly regulated apoptotic pathways. Out of the 70 molecular functions (MF, GO-term) significantly enriched in LH + DDP group, THBS1 overlapped in 11 molecule binding functions. THBS1 was the top one up-gene in the two pathways of the Focal adhesion/ECM-receptor interaction pathway network in LH + DDP group by KEGG. ECM receptor interaction pathway and focal adhesion pathway control many important cellular activities, such as apoptosis, proliferation, differentiation, adhesion, migration and motility. THBS1 seems to be the critical gene involving in the enhancement of anticancer efficacy in LH + DDP group.

LAPTM5 (lysosomal-associated protein multispanning transmembrane 5) is a membrane protein which localizes to intracellular vesicles. Expression level of LAPTM5 is frequently decreased in numerous cancer cell lines [33]. LAPTM5 overexpression in cancer cells induces lysosomal cell death through lysosomal destabilization [34]. Low expression of LAPTM5 in cancer patients is correlated with poor prognosis. Inactivation of LAPTM5 is associated with tumorigenesis in human cancers [35]. Expression of LAPTM5 was upregulated by LH, DDP and LH + DDP, which favored the anticancer activity in these groups. LAPTM5 expression level in LH + DDP group was much higher than that in LH group and in DDP group, which is consistent with the enhancement of anticancer efficacy in combination group. LAPTM5 overexpression and the consequently lysosomal cell death due to lysosomal destabilization, provided reasonable explanation for the large fragment peak in front of G0/G1 in cell cycle detection by flow cytometry.

TNFAIP3 (tumor necrosis factor α-induced protein 3) is a deubiquitinating enzyme which belongs to zinc finger protein family. Knock out of TNFAIP3 enhances proliferation and invasion of lung cancer cells [36]. Downregulation of TNFAIP3 is associated with distant metastasis and poor prognosis in cancer patients [37]. TNFAIP3 overexpression alleviates migration and invasion. Knockout of TNFAIP3 attenuates the ability of proliferation, invasion and migration of cancer cells in vitro [38]. Our results showed TNFAIP3 overexpressed in LH + DDP group and overlapped in 14 of 20 significantly enriched apoptotic pathways. LogFC of TNFAIP3 was 0.330, 1.095 and 1.714 in LH group, DDP group and LH + DDP group respectively. Expression level of TNFAIP3 was further enhanced by LH + DDP by microarray data, which was consistent with the enhancement of the anticancer efficacy in LH + DDP group.

Jak-STAT signaling pathway, which affects the proliferation, survival and invasion of cancer cells, is a favorite target for cancer therapy and drug development [39]. Targeting of Jak-STAT pathway is now one of the most hopeful therapeutic strategies in cancer therapy [40]. In the present study, Jak-STAT / Cytokine-cytokine receptor interaction pathway network was the common network significantly enriched in LH group, DDP group and LH + DDP group by KEGG. Interleukin 24 (IL24) was the top one overlapped differential gene in the two pathways in this network. IL24 overexpressed in LH group and LH + DDP group, and the expression level of IL24 was further enhanced in LH + DDP group. IL24 is a cytokine, belonging to the IL10 subfamily. Upregulation of IL24 is able to induce apoptosis selectively in many cancers and xenograft models [41, 42]. IL24 overexpression inhibits colony formation and cell proliferation, and induces apoptosis and cell cycle arrest of hepatocellular carcinoma cells. IL24 is a broad-spectrum anticancer gene, which is able to induce toxic autophagy and apoptosis selectively in transformed cells and contributes to terminal cell differentiation [43]. IL24 regulates many cancer-associated pathways and oncogenes, including apoptosis activation and cell cycle regulation pathways [44]. Overexpression and further upregulation of IL24 in LH + DDP group was likely associated with the enhancement of anticancer efficacy, by regulation of Jak-STAT/Cytokine-cytokine receptor interaction pathway network.

SRSF6 regulates alternative splicing. Upregulation of SRSF6 enhances metastasis and proliferation of cancer cells in vitro and in vivo [45]. SRSF6 is often upregulated in colorectal cancer and associated with worse prognosis. SFPQ is an RNA binding protein. SFPQ expression level in human hepatocellular carcinoma is much higher than that in adjacent tissues [46]. The survival rate of cancer patients with low SPFQ expression is significantly higher than that of patients with high SPFQ expression. In our study, SRSF6 and SFPQ were downregulated in LH group, DDP group and LH + DDP group. The expression levels of SRSF6 and SFPQ in LH + DDP group were lower than those in DDP group and in LH group. SRSF6 and SFPQ overlapped in 1 and 3 significantly regulated apoptotic pathways respectively (BP, GO-term). Low expression level and further downregulation of SRSF6 and SFPQ in LH + DDP group supported the enhancement of anticancer efficacy.

NCOA5 modulates ERα-mediated transcription and is correlated with the progression of various malignancies. Overexpression of NCOA5 is significantly associated with progression and worse prognosis in human breast cancer, and patients with low NCOA5 expression level have significantly higher overall survival [47]. Expression level of NCOA5 in colorectal carcinoma (CRC) tissues is much higher than that in adjacent tissues [48]. Suppression of NCOA5 notably inhibits proliferation, invasion and migration of CRC cells in vitro. Knockdown of NCOA5 also suppresses growth of CRC xenograft tumors in vivo. Upregulation of NCOA5 promotes proliferation, invasion and migration of CRC cells. In this study, NCOA5 was downregulated by LH, DDP and LH + DDP. REL of NCOA5 in LH + DDP group was significantly less than that in LH group and in DDP group. LH + DDP further reduced the expression level of NCOA5 in cancer cells, which is consistent with the enhancement of anticancer efficacy in LH + DDP group.

Although LH concentration used in the experiments is much higher than the serum or tissue concentration of levofloxacin when LH is used for treatment of infectious diseases, 25–200 µg/ml of LH concentration used in the study is practical when LH is used for the treatment of malignant ascites or pleural fluid. LH concentration in malignant ascites or pleural fluid could be much higher than 200 µg/ml when it is used by thoracic perfusion or intraperitoneal perfusion at 500 mg/day or 750 mg/day. We find that levofloxacin at the concentration of 25 μg/ml shows significant antiproliferation activity in all cancer cell lines in the cell viability assay [13] (Supplementary Fig. S1A). Baum et al. report that the tissue concentration of levofloxacin from 500 mg/day levofloxacin treatment is close to the 25 μg/ml dose [49]. Concentration of levofloxacin in blood and tissues increases with the increasing of levofloxacin dose [49, 50]. Therefore, we deduce that levofloxacin at the concentration of 25 μg/ml or a higher concentration in the blood or tissues of cancer patients can enhance the anticancer efficacy of chemotherapeutic drugs although this speculation needs for further verification.

As all current chemotherapeutic drugs used in cancer treatment have severe toxic side effects and yet accepted by patients, higher levofloxacin doses than 750 mg/day, if it is warranted to be allowable in cancer patients despite its potential side effects, will lead to much higher levofloxacin concentration in blood and in cancer sites. It is therefore possible that the maximal 750 mg/day dose of levofloxacin used for the treatment of bacterial infections in human or higher allowable dose of levofloxacin used for cancer therapy will provide a much more effective local levofloxacin concentration at tumor sites for the combined chemotherapy. Interestingly, in vivo combined administration of 50 mg/kg levofloxacin in our xenograft study suggests that extremely high dose may not be required to enhance the tumor growth inhibition effect of chemotherapeutic drugs in vivo.

## Conclusion

Combination of levofloxacin and chemotherapeutic drugs enhanced anticancer efficacy both in vitro and in vivo, which was associated with the further regulation of THBS1, TNFAIP3, LAPTM5, PI3, IL24 and NCOA5, SFPQ, SRSF6. Targeting of Focal adhesion/EMC-receptor interaction/Jak-STAT/Cytokine-cytokine receptor interaction pathway network was correlated to the enhancement. With additional benefit to cancer patients for treatment or prophylaxis of an infectious syndrome, levofloxacin can benefit cancer therapy either as a broad-spectrum anticancer agent or as a chemosensitizer of chemotherapeutic drugs in combination chemotherapy.

## Supplementary Information

Additional file1: Figure S1. Effect of LH on the cell viability in different cancer lines and the REL of the differential up-genes regulated by LH. A OD of different cancer cells treated with different concentrations of LH [13]. B OD of CNE2 cells treated with different concentrations of 5-Fu in no LH medium (LH: 0 μg/ml) and in LH medium (LH: 200 μg/ml). C REL of the targeted differential genes upregulated by LH [13]. Figure S2 Top ten cell components, biological pathways and molecular functions significantly enriched in LH + DDP group. A Upgene-Sig-Go. B Downgene-Sig-Go. C Upgene_Sig_GoEnrichment. D Downgene_Sig_GoEnrichment. Table S1 Apoptotic pathways significantly modulated by the upregulated differential genes. Table S2 Apoptotic pathways significantly modulated by the downregulated differential genes. Table S3 Signaling pathways significantly regulated by up-genes in KEGG enrichment. Table S4 Signaling pathways significantly regulated by down-genes in KEGG enrichment. Table S5 Primer sequences used for RT–qPCR assay. (DOCX 593 KB)

## Data Availability

All data are available in the main text. Raw data of microarray gene expression profiling and RT–qPCR can be provided on requests.
