# Supplementary material for: Combination of levofloxacin and cisplatin enhances anticancer efficacy via co-regulation of eight cancer-associated genes
Source: Discov Oncol. 2022 Aug 19;13:76. doi: 10.1007/s12672-022-00541-x (PMC9391551; doi:10.1007/s12672-022-00541-x)
Supplement: Supplementary file 1 — Additional file1: Figure S1. Effect of LH on the cell viability in different cancer lines and the REL of the differential up-genes regulated by LH. A OD of different cancer cells treated with different concentrations of LH [13]. B OD of CNE2 cells treated with different concentrations of 5-Fu in no LH medium (LH: 0 μg/ml) and in LH medium (LH: 200 μg/ml). C REL of the targeted differential genes upregulated by LH [13]. Figure S2 Top ten cell components, biological pathways and molecular functions significantly enriched in LH + DDP group. A Upgene-Sig-Go. B Downgene-Sig-Go. C Upgene_Sig_GoEnrichment. D Downgene_Sig_GoEnrichment. Table S1 Apoptotic pathways significantly modulated by the upregulated differential genes. Table S2 Apoptotic pathways significantly modulated by the downregulated differential genes. Table S3 Signaling pathways significantly regulated by up-genes in KEGG enrichment. Table S4 Signaling pathways significantly regulated by down-genes in KEGG enrichment. Table S5 Primer sequences used for RT–qPCR assay. (DOCX 593 KB) [file 12672_2022_541_MOESM1_ESM.docx]

**Fig. S1** Effect of LH on the cell viability in different cancer lines and the REL of the differential up-genes regulated by LH. **A** OD of different cancer cells treated with different concentrations of LH [13]. **B** OD of CNE2 cells treated with different concentrations of 5-Fu in no LH medium (LH: 0 μg/ml) and in LH medium (LH: 200 μg/ml). **C** REL of the targeted differential genes upregulated by LH [13].

**Fig. S2** Top ten cell components, biological pathways and molecular functions significantly enriched in LH+DDP group. **A** Upgene-Sig-Go. **B** Downgene-Sig-Go. **C** Upgene_Sig_GoEnrichment. **D** Downgene_Sig_GoEnrichment.

**Table S1.** Apoptotic pathways significantly modulated by the upregulated differential genes.

| goID | goDescription | LH | DDP | LH+DDP |
| --- | --- | --- | --- | --- |
| GO:2001236 | regulation of extrinsic apoptotic signaling pathway | √ | √ | √ |
| GO:2001240 | negative regulation of extrinsic apoptotic signaling pathway in absence of ligand | √ | × | √ |
| GO:2001239 | regulation of extrinsic apoptotic signaling pathway in absence of ligand | √ | × | √ |
| GO:2001234 | negative regulation of apoptotic signaling pathway | √ | × | √ |
| GO:0097191 | extrinsic apoptotic signaling pathway | √ | × | √ |
| GO:1904035 | regulation of epithelial cell apoptotic process | √ | × | √ |
| GO:1904019 | epithelial cell apoptotic process | √ | × | √ |
| GO:2001233 | regulation of apoptotic signaling pathway | √ | × | √ |
| GO:2000352 | negative regulation of endothelial cell apoptotic process | √ | × | √ |
| GO:1904036 | negative regulation of epithelial cell apoptotic process | √ | × | √ |
| GO:1902042 | negative regulation of extrinsic apoptotic signaling pathway via death domain receptors | √ | × | √ |
| GO:2000351 | regulation of endothelial cell apoptotic process | √ | × | √ |
| GO:0072577 | endothelial cell apoptotic process | √ | × | √ |
| GO:1902041 | regulation of extrinsic apoptotic signaling pathway via death domain receptors | √ | × | √ |
| GO:0032496 | response to lipopolysaccharide | √ | × | × |
| GO:0097192 | extrinsic apoptotic signaling pathway in absence of ligand | √ | × | × |
| GO:0097283 | keratinocyte apoptotic process | √ | × | × |
| GO:1902172 | regulation of keratinocyte apoptotic process | √ | × | × |
| GO:0003278 | apoptotic process involved in heart morphogenesis | × | √ | × |
| GO:2000427 | positive regulation of apoptotic cell clearance | × | √ | × |
| GO:2000425 | regulation of apoptotic cell clearance | × | √ | × |
| GO:2001241 | positive regulation of extrinsic apoptotic signaling pathway in absence of ligand | × | √ | × |
| GO:0043280 | positive regulation of cysteine-type endopeptidase activity involved in apoptotic process | × | √ | × |
| GO:2001237 | negative regulation of extrinsic apoptotic signaling pathway | × | × | √ |
| GO:0001783 | B cell apoptotic process | × | × | √ |
| GO:0008625 | extrinsic apoptotic signaling pathway via death domain receptors | × | × | √ |
| GO:0043277 | apoptotic cell clearance | × | × | √ |
| GO:0003278 | apoptotic process involved in heart morphogenesis | × | × | √ |
| GO:0043652 | engulfment of apoptotic cell | × | × | √ |

√: regulated. ×: unregulated.

**Table S2.** Apoptotic pathways significantly modulated by the downregulated differential genes.

| goID | goDescription | LH | DDP | LH+DDP |
| --- | --- | --- | --- | --- |
| GO:2000675 | negative regulation of type B pancreatic cell apoptotic process | √ | × | √ |
| GO:1902177 | positive regulation of oxidative stress-induced intrinsic apoptotic signaling pathway | × | × | √ |
| GO:2000674 | regulation of type B pancreatic cell apoptotic process | × | × | √ |
| GO:0097050 | type B pancreatic cell apoptotic process | × | × | √ |
| GO:0072332 | intrinsic apoptotic signaling pathway by p53 class mediator | × | √ | × |
| GO:0042771 | intrinsic apoptotic signaling pathway in response to DNA damage by p53 class mediator | × | √ | × |

√: regulated. ×: unregulated.

**Table S3.** Signaling pathways significantly regulated by up-genes in KEGG enrichment.

| pathID | pathDescription | LH | DDP | LH+DDP |
| --- | --- | --- | --- | --- |
| hsa04060 | Cytokine-cytokine receptor interaction | √ | √ | √ |
| hsa04630 | Jak-STAT signaling pathway | √ | √ | √ |
| hsa05020 | Prion diseases | √ | × | √ |
| hsa00140 | Steroid hormone biosynthesis | √ | × | √ |
| hsa00980 | Metabolism of xenobiotics by cytochrome P450 | √ | × | √ |
| hsa05140 | Leishmaniasis | √ | × | √ |
| hsa04640 | Hematopoietic cell lineage | √ | × | √ |
| hsa05323 | Rheumatoid arthritis | √ | × | √ |
| hsa04380 | Osteoclast differentiation | √ | × | √ |
| hsa04710 | Circadian rhythm | √ | × | × |
| hsa05332 | Graft-versus-host disease | √ | × | × |
| hsa04940 | Type I diabetes mellitus | √ | × | × |
| hsa04210 | Apoptosis | √ | × | × |
| hsa04010 | MAPK signaling pathway | √ | × | × |
| hsa05142 | Chagas disease (American trypanosomiasis) | √ | × | × |
| hsa04142 | Lysosome | × | √ | × |
| hsa05340 | Primary immunodeficiency | × | √ | √ |
| hsa04512 | ECM-receptor interaction | × | × | √ |
| hsa04610 | Complement and coagulation cascades | × | × | √ |
| hsa05144 | Malaria | × | × | √ |
| hsa05150 | Staphylococcus aureus infection | × | × | √ |
| hsa05146 | Amoebiasis | × | × | √ |
| hsa04621 | NOD-like receptor signaling pathway | × | × | √ |
| hsa04510 | Focal adhesion | × | × | √ |
| hsa04146 | Peroxisome | × | × | √ |
| hsa05222 | Small cell lung cancer | × | × | √ |
| hsa00524 | Butirosin and neomycin biosynthesis | × | × | √ |

√: regulated. ×: unregulated.

**Table S4.** Signaling pathways significantly regulated by down-genes in KEGG enrichment.

| pathID | pathDescription | LH | DDP | LH+DDP |
| --- | --- | --- | --- | --- |
| hsa00510 | N-Glycan biosynthesis | √ | × | √ |
| hsa03440 | Homologous recombination | √ | × | × |
| hsa03020 | RNA polymerase | √ | × | × |
| hsa00230 | Purine metabolism | √ | × | × |
| hsa00240 | Pyrimidine metabolism | √ | × | × |
| hsa04620 | Toll-like receptor signaling pathway | √ | × | × |
| hsa00310 | Lysine degradation | √ | × | × |
| hsa00970 | Aminoacyl-tRNA biosynthesis | × | √ | × |
| hsa04110 | Cell cycle | × | √ | × |
| hsa05322 | Systemic lupus erythematosus | × | √ | √ |
| hsa00750 | Vitamin B6 metabolism | × | √ | √ |
| hsa04142 | Lysosome | × | × | √ |
| hsa04742 | Taste transduction | × | × | √ |

√: regulated. ×: unregulated.

**Table S5.** Primer sequences used for RT-qPCR assay.

| Gene Name | Forward | Reverse |
| --- | --- | --- |
| LAPTM5 | ACCTGCTGCTGCTTCAATGT | GGCCACCTCTACTGAGTGCT |
| THBS1 | ATTTCACCGCCTACAGATGG | GGTCCTGAGTCAGCCATGAT |
| PI3 | AGCAGCTTCTTGATCGTGGT | GACCTTTGACTGGCTCTTGC |
| TNFAIP3 | TGGCTGAACAAGTCCTTCCT | TCCAGTGTGTATCGGTGCAT |
| SFPQ | CATGATGGGAAGTGACATGC | TCCCTCTACCATATCCTGCTG |
| NCOA5 | GGATACCCTGATCCAGAGTGG | CAGTAATGCCTCTGGTAAGATCC |
| SRSF6 | CGCCTCCTCGAAGTAGACCT | AGAGCTCCTTGCCGTTCAG |
